# Knowledge, attitudes and beliefs about acute coronary syndrome among patients diagnosed with acute coronary syndrome, Addis Ababa, Ethiopia

**DOI:** 10.1186/s12872-022-02893-2

**Published:** 2022-10-14

**Authors:** Lemlem Demisse, Bekele Alemayehu, Adamu Addissie, Aklilu Azazh, Rebecca Gary

**Affiliations:** 1 College of Health Sciences, Addis Ababa University, Addis Ababa, Ethiopia; 2 Nell Hodgson Woodruff School of Nursing, Emory University, Atlanta, Georgia

**Keywords:** Knowledge, Attitudes, Beliefs, Acute coronary syndrome, Ethiopia

## Abstract

**Background:**

Acute coronary syndrome (ACS) morbidity and mortality are rising in low- and middle-income countries, including Ethiopia. The shift in health-care resources from communicable diseases to chronic conditions has created formidable health-care challenges.

**Objective:**

The objective of this study was to examine the knowledge, attitudes and beliefs among ACS patients.

**Methods:**

A cross-sectional design was used to enroll participants admitted to one of 3 emergency units in Addis Ababa, Ethiopia. Knowledge, attitudes and beliefs about ACS was measured using modified ACS response index questionaries.

**Results:**

Participant’s (N = 330) mean age was 57.9 ± 14.1, majority male (n = 219, 66.36%). Half of the study participants have inadequate Knowledge (n = 147, 44.6%), unfavorable attitudes (n = 152, 46%), and belief (n = 153, 46.4%) about ACS symptoms even after being diagnosed and treated in the emergency unit. The most frequently recognized ACS symptoms were chest discomfort (n = 274, 83%), fatigue (n = 267, 80.9%) and chest pain (n = 266, 80.6%) while Jaw pain (n = 101, 30%) neck pain (n = 146,44.2%), were less often recognized. Nearly two thirds of the participants (n = 214, 65%) would not prefer to use emergency medical services (EMS) to come to the hospital. Factors associated with adequate knowledge were age < 45 (AOR = 2.16, CI (1.1–4.0) p = 0.014), and female sex (AOR = 2.7, CI (1.5–4.4) p = 0.001) and diabetics (AOR = 1.9, (1.18–3.0) p = 0.008). Meanwhile, lack of formal education (AOR = 6.7, CI (3.1–14) p < 0.001) and unemployment (AOR = 2.0, CI (1.1–3.8) p = 0.021) were associated with unfavorable attitude. In addition, lack of social support (AOR = 1.9, (1.17–3.0) p = 0.009) and unfavorable attitude (AOR = 2.1, CI (1.3–3.4) p = 0.001) were significantly associated with unfavorable belief.

**Conclusion:**

Despite receiving treatment for ACS in an emergency unit, roughly half of participants did not have adequate knowledge, favorable attitude and belief towards ACS. This elucidates there is significant communication gap between the health care providers and patients. The study findings stipulate there is a need to provide health awareness campaigns using different media outlet with special attention to the uneducated and unemployed groups. Furthermore, most participants were less likely to utilize emergency medical service, which should be further investigated and addressed.

## Introduction

A marked reduction in coronary artery blood flow potentially led to a range of conditions known as acute coronary syndrome (ACS). Stable angina (UA), ST-segment elevation myocardial infarction (STEMI), or non-ST segment elevation myocardial infarction (NSTEMI) are the most frequent symptoms [1]. Although ACS rates are declining in high-income countries, but are rapidly growing in LMICs, largely because of urbanization and westernization [2]. Despite to previous studies that claimed rheumatic heart disease was the most prevalent cause of cardiovascular disease (CVD), coronary heart disease now tops the list of causes of morbidity among Ethiopians [3]. According to the World Health Organization (WHO), AMI and stroke accounted for 85% of the estimated 17.9 million CVD deaths in 2015, accounting for 31% of all global deaths [4]. Despite the fact that nationwide CVD studies are scarce in Ethiopia, evidence from global burden data in 2017 indicates that CVD is the leading cause of death [3]. Elevated blood pressure, obesity, dyslipidemia, physical inactivity, smoking, and substance use such as tobacco were identified as the most common risk factors for CVD in Ethiopia.[5–7].

It is well known that detecting ACS symptoms early and initiating reperfusion therapy within 3- hours is associated with a significant reduction in mortality [8]. Patients should have adequate knowledge, beliefs, and attitudes about ACS symptoms in order to seek treatment early. Knowledge of ACS symptoms and risk factors is linked to more positive attitudes and beliefs about early treatment. [9]. It has been reported that a lack of knowledge contributes to ACS treatment delays [10–13]. Evidence suggests that ACS educational interventions lead to improved knowledge and earlier treatment seeking, which improves clinical outcomes [9, 14, 15]. The concepts of knowledge, attitudes, and beliefs are intricate and distinct. Individual knowledge is the capacity for comprehending information or facts acquired through training or experience. An attitude is a sentiment or view, but a belief is a sense of conviction about something's reality [16]. The literature often used these terms interchangeably, and the differences among them are not always explicitly defined [17]. Timely identification of ACS symptoms is crucial for prompt receipt of advanced care, such as percutaneous coronary interventions (PCI), as per research [18, 19]. While multiple researches of ACS symptom knowledge have been conducted in middle to high income countries, data in Africa is limited. The purpose of the study was to examine patients' knowledge, attitudes, and beliefs about ACS symptoms who were admitted to three emergency units in Addis Ababa, Ethiopia.

## Methods

### Design

A cross-sectional study design was employed to collect data among ACS patient from November 2019 to December 2020.

### Study participants and setting

Participants who were diagnosed with ACS and had presented within 48–72 h to the emergency units (EU) of three tertiary care hospitals (two public tertiary and one private hospital) in Addis Ababa, Ethiopia. These hospitals were selected because visited by large volume of cardiac patients, availability of advanced cardiovascular services such as cardiac catheterization and reperfusion therapy [20]. These hospitals also have a coronary care unit (CCU) for post-surgery and critically ill ACS patients, intensive care unit (ICU), well-established emergency admission unit and, emergency physician for acute cardiac emergencies.

### Sampling

All consecutive patients with a diagnosis of ACS and met eligibility criteria were recruited from the EU. The eligibility criteria were: (1) 18 years or older, (2) ability to understand and speak Amharic or English, (3) a confirmed diagnosis of ACS documented in medical records (as evidenced by abnormal cardiac bio-markers such as elevated cardiac enzyme (Troponin -I Creatine Kinase—MB (CK -MB) or dynamic electrocardiogram (EKG) changes indicative of STEMI, NSTEMI or diagnosed by symptomatology (chest pain, shortness of breath, dizziness, or light headedness), (4) ability to recall the time symptoms started and events prior to hospital admission and, (5) hemodynamically stable as confirmed by stable vital signs, free of chest pain or discomfort at the time of data collection. Exclusion criteria included*:* (1) cognitive impairment or inability to understand or communicate, (2) major psychiatric disorder, (3) critically ill, (4) mechanically ventilated, (5) terminal illness or (6) multiple diagnoses that complicated symptom recognition. Sample size was estimated using two population proportion formulas using StatCalc of Epi-info version 7.2.2.6 statistical software. To get the representative sample the proportion of acute coronary syndrome who perceived their symptoms as cardiac origin is 28.6% and Odds ratio of 1.91 [11]. Out of 375 invitees, only 330 agreed to participate (88%). The level of significance for statistical analysis was set as 0.05, 95% confidence interval (CI) and power of 0.8 [21].

### Data collection procedure

Ethical approval was obtained from Institutional Review Board (IRB) (No.078/19/Nursing) of Addis Ababa University, College of Sciences. Data were collected by face-to-face interview and review of participants medical records. Prior data collection written informed consent was obtained from eligible participants and also informed that participation is voluntary and could withdraw any time without any influence on their treatment. Additionally, participants were notified they may not directly benefit from the study, but the information may be useful for other patients with the same condition in the future. Following informed consent data was collected by nurses working in each EU after they received two-days training.

### Data collection instrument

The modified ACS Response Index questionnaires (ACSRI) were used to measure knowledge, attitudes, and beliefs about ACS [22], and has been widely used in the ACS population. The internal consistency has been documented as 0.81 for knowledge, 0.76 attitudes and 0.74 beliefs [22]. In the current study, internal consistency measurement was 0.76 for knowledge, 0.81 for attitudes and 0.64 for beliefs. The Amharic version ACSRI questionnaire was not previously tested in an Ethiopian population hence, was pilot-tested in 10 patients not include in the study. The level of knowledge was measured on a dichotomous scale. From a list of 15 predetermined ACS symptoms, patients were asked to correctly identify the symptoms that could indicate to a heart attack. Additionally, six distractor symptoms were also included to assess and account for the likelihood that participants would select "yes" for each of the items. The total combined knowledge score was 21. Using the mean score (14.06), participants were classified as having adequate or inadequate knowledge. Attitudes and beliefs were measured using a 4-point Likert scale. The attitudes scale had five items, which documented the patients’ attitudes on their ability to recognize symptoms and initiate appropriate help-seeking behavior. The total score ranges from 5 to 20 (5- indicating poor recognition and less likelihood of activating help while 20-indicating the participant would identify ACS symptoms and promptly seek help). The belief subscale has 5 items that assess the participants beliefs and identifies actions for future ACS symptoms. The scores from this subscale ranges from 5 to 20 with higher scores representing beliefs that they would take action for a future ACS event.

### Data analysis

Data were entered in to Epi data and exported to STATA version 17.1 statistical package for analysis. The data generated from medical records and self -report were screened for accuracy and completeness. The distribution of continuous data was checked using box-plots and histogram. Descriptive statistics mean ± SD (mean and standard deviation) were used for continuous variables or frequency (%) for other variables. The knowledge, attitudes and beliefs score were divided in two group at the (mean score) those participants who scored greater than or equal to the mean were grouped as having adequate knowledge, favorable attitude and beliefs. Assumption of logistic regression was checked for linearity of the logit of dependent variable with continuous independent variable, and multicollinearity. Also, model fitness was assessed prior to data analysis and no major violation was identified. Multiple logistic regression was used to identify the independent predictors of adequate versus inadequate knowledge, favorable versus unfavorable attitudes and beliefs related to socio-demographic, clinical characteristics and psychosocial variables.

## Results

### Demographic characteristics

Out of three hundred seventy-five participants only 330 agreed to participate (88%, response rate). The mean ± SD age of the study participants was 57 ± 14 years and majority were male (n = 219, 66.3%). Most lived in urban areas (n = 228, 69%) and were college/university graduates (n = 138, 41.8%). The sociodemographic characteristics are presented in Table 1.

**Table 1:** Socio-demographic characteristics of participants with acute coronary syndrome admitted in selected private and public hospitals of Addis Ababa, Ethiopia from 2019/20 year(n = 330)

Variables	Frequency/mean (SD)	Percent
Age (years)	56 ± 13	
*Sex*		
Male	219	66.36
Female	111	34.64
*Residence*		
Urban	228	69.09
Rural	102	30.91
*Mode of transport*		
Ambulance	49	14.85
non-ambulance	281	85.15
*Marital status*		
Never married	29	8.79
Ever Married	301	91.21
*Education level*		
No formal education	80	24.24
Never educated beyond secondary school	112	33.94
College and above	138	41.82
*Occupation*		
Employed	138	41.82
Unemployed	135	40.91
Retired	57	17.27
*Exposure to Mass media*		
Television	294	89.09
Radio	144	43.64
*History*		
Hypertension	185	56
Diabetes	180	54
Smoking	71	21

### Description of knowledge, attitudes and beliefs

#### Knowledge

Participants mean knowledge score was 14.06 ± 3.9 out of a total score of 21. Using mean score as the cut-off value, (n = 183, 55.4%) participants were found to have adequate knowledge. Exploring knowledge of individual ACS symptoms, majority of the participants described chest discomfort /heaviness (83%) as a symptom of a heart attack. As seen in Table 2, participants mistakenly thought that distractor symptoms such arm paralysis (67.2%), numbness or tingling in the arm (63%) slurred speech (62%) and headache (58%) were ACS symptoms.

**Table 2:** Correct and incorrect responses of ACS response index questions on individual acute coronary syndrome symptoms (n = 330) of participants admitted at private and public hospital of Addis Ababa, Ethiopia

Symptoms	n (%)
Chest discomfort/heaviness	**274 (83.03)**
Weakness/Fatigue	**267(80.91)**
Chest pain pressure/tightness	**266(80.61)**
Shortness of breath	261(79.09)
Dizziness/lightheaded	246(74.55)
Arm pain or shoulder pain	238(72.1)
Sweating	234(70.9)
*Arm paralysis	222(67.27)
Numbness/tingling in the arm	209(63.33)
*Slurred/speech	205(62.12)
*Headache	194(58.79)
Heart burn/indigestion/stomach problem	173(52.42)
*Lower abdominal pain	171(51.82)
*Back pain	168(50.91)
*Cough	162(49.09)
Nausea/vomiting	159(48.1)
Loss of consciousness	150(45.45)
Neck pain	146(44.2)
Pale ashen or color change/palpitation/rapid heart rate	134(40.61)
Jaw pain	101(30.6)

ACS symptoms in bold are those identified by the majority of study participants

Values: frequency (%); symptoms with “*” are distractors symptoms

#### Attitude

**Fig. 1:**
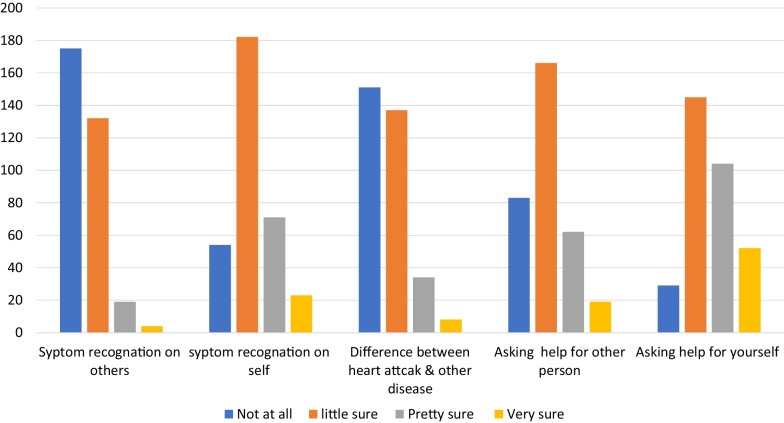
Participant’s response on attitude sub scale of the ACS Response Index who were admitted at selected private and public hospital of Addis Ababa, Ethiopia

The participants mean score on attitude towards ACS was 10.0 ± 2.96 out of a total of 20. Using the mean score as the cutoff of value, 178 (53.9%) of the participants were found to have favorable attitude. In addition, the majority of participants were little sure of their ability to recognize ACS symptoms (N = 182, 55.1%). Only (n = 104, 31.5%) of participants indicated they were certain they would get help if they experienced future ACS symptoms. (Fig. 1).

#### Belief

The mean belief score was 11.59 ± 3.15 out of total score of 20. Using the mean score as the cut-off value, (177, 53.64%) had favorable beliefs. Forty-four percent (n = 148) of the participants agreed that they would go to a hospital immediately if they were experiencing chest pain even if they were unsure of the origin of the pain. Thirty five percent (n = 116) would prefer for someone to drive them to the hospital rather than using an ambulance Table 3.

**Table 3:** Participants response on belief subscale of the ACS Response Index who were admitted at selected private and public hospital of Addis Ababa, Ethiopia. (N = 330)

Variable	Strongly agree	Agree	Disagree	Strongly disagree
I would be embarrassed to go to the hospital if I thought I was having a heart attack but not	44(13.33)	94(28.48)	118(35.76)	74(22.42)
If I thought I was having a heart attack, I would wait until I was very sure before going to the hospital	70(21.21)	111(33.64)	90(27.27)	59 (17.88)
If I thought I was having a heart attack, I would rather have someone drive me to the hospital than have an ambulance come to my home	112(33.94)	116(35.15)	60(18.18)	42(12.73)
B/C of the cost of medical care, I would want to be absolutely sure I was having a heart attack before going to the hospital	77(23.33)	111(33.64)	80(24.24)	62(18.79)
If I’m having chest pain and I’m not very sure if it’s a heart attack, I should go to the hospital	99(30.00)	148(44.85)	59(17.88)	24(7.27)

### Factors associated with knowledge, attitude and belief of ACS symptoms

Multivariable logistic regression was used to identify independent predictors of knowledge level, attitudes and beliefs about ACS.

#### Knowledge

The following predicator variables such as age, gender, place of residence, history of diabetes, family history of premature death and social support were entered into a logistic regression model. However, only age, gender and patient with diabetes where significantly associated with level of knowledge. Participants with an age less than or equal to 45 years were 2.16 times more likely to have adequate knowledge than those above 56 years age (AOR = 2.16, 95%CI: [1.16, 4.0], p = 0.014). Females were also 2.7 more likely to have an adequate knowledge of ACS than males (AOR = 2.7, 95% CI; [1.59, 4.44], p =  < 0.001). In addition, participants with diabetes comorbidity were 1.9 times more likely to identify ACS symptoms compared to those with no diabetes (AOR = 1.9, 95% CI; [1.18, 3.0], p = 0.008) Table 4a.

**Table 4:** Predictors of knowledge, attitude and belief of participants towards acute coronary syndrome

Variables	AOR with (95% CI)	P-value
4a. Knowledge		
**Age:** < = 45years^a^	2.16 (1.16, 4.00)	0.014*
**Gender:** Female	2.7(1.59, 4.44)	< 0.001***
**Diabetes**^**b**^	1.9 (1.18, 3.0)	0.008**
4b. Attitude		
**Level of education**^**c**^		
Never attend formal education	0.14 (0.06, 0.31)	< 0.001 ***
Primary/secondary education	0.44 (0.24, .81)	0.009**
**Occupation**^**d**^: Unemployed	0.47 (0.25, 0.89)	0.021 *
**Exposure to mass media***: Radio*	2.13 (1.23, 3.68)	0.006**
4c. Belief		
**Adequate social support**^**e**^	1.76(1.03, 3.01)	0.038*
**Living with others** *(non- family)*^***f***^	2.55(1.12, 5.83)	0.026*
**Adequate social support**	1.96(1.24, 3.16)	0.004*
Unfavorable attitudes^g^	0.45(0.28, 0. .71)	0.001**

AOR; adjusted odds ratio reported; confidence interval in parenthesis

Variable in bold are factors that have statsically significant assocation with outcome varable

*p < 0.05, **p < 0.01, ***p < 0.001

^a^Age group compare with < 56 years, ^b^Diabetes with non-diabetes, ^c^All level of education with college diploma or higher, ^d^Employment status un employment compares with Employed, ^e^social support adequate with inadequate, ^f^Living arrangement live with children with non-family member, ^g^attitude favorable versus unfavorable

#### Attitude

Regarding attitudes five predicator variables (education, occupation, exposure to mass media, living arrangement, social support) in the model were statistically significant predictors of attitude towards ACS. Those with lower educational levels were 86% less likely to have favorable attitudes towards ACS symptoms (AOR = 0.14 95% CI: [0.06, 0.31], p < 0.001). Unemployed participants were 53% less likely to have favorable attitudes comparing to those employed (AOR = 0.47, 95% CI [0.25, 0.89], p = 0.021). In addition, the odds of a favorable attitude regarding ACS among participants with exposure to mass media was 1.6 times higher than those without media exposure. Those with adequate social support had a 1.76 times more favorable attitude than those who had inadequate social support (AOR = 1.76, 95% CI [1.03, 3.01], p = 0.038). Moreover, participants who reported to live with distant family members were found to have 2.55 times favorable attitudes towards ACS. (AOR = 2.55,95% CI [1.12, 5.83], p = 0.026) Table 4b.

#### Belief

Those who perceived themselves as having good social support were 1.9 more likely to have favorable belief towards ACS symptoms (AOR = 1.9, 95% CI (1.24, 3.16), p = 0.004). Conversely, those participants with unfavorable attitudes score were 55% times more likely to have unfavorable beliefs towards ACS symptoms (AOR = 0.45, 95% CI (0.28, 0. 71), p = 0.001) Table 4c.

## Discussion

Our findings are among the first to describe the knowledge, attitudes and beliefs among Ethiopian patients hospitalized for ACS. The findings showed that slightly more than half of the participants were able to correctly identify some, but not all ACS symptoms which is similar to other reports [23, 24]. Albarquoin et al. 2016 identified that half of the patients (n = 285, 58%) demonstrated high knowledge of ACS and the majority (n = 476, 98%) recognized at least one ACS symptom. In addition, a study that was conducted in 3 countries (United States, Australia and New Zealand) also reported comparable results with 56% of participants having adequate knowledge of ACS symptoms [24]. An Irish study (n = 964, 49.5%) demonstrated a slightly lower level of ACS knowledge compared with the present study but the majority were able to recognize chest pain/pressure as a symptom (98.9%) [9]. Similarly, in the present study most participants identified chest discomfort/ heaviness (83.6%) as a symptom of ACS. Conversely, a Pakistan study reported that 81% of 720 participants failed to recognize symptoms and only 6% were able to identify one or more symptoms of ACS [25]. This finding was also supported by Noureddine et.al, 2020 where only 13 out of 50 participants scored greater than 70% in the knowledge questions. However, greater than 85% recognized typical symptoms such as chest pain and sweating [26]. Suboptimal knowledge related to cardiovascular disease was also reported by Negesa et al., (n = 132, 46%) from Ethiopia [27]. The result is slightly lower than the report from the current study. Based on the literature, we found considerable variability concerning the identification of ACS symptom knowledge. There are several potential explanations including differences in study settings, socioeconomic status, health literacy level and the level of exposure to mass media campaigns. Many of our findings about our participants' sociodemographic characteristics are consistent with research done in Ethiopia and other LMICs. When compared to high-income countries, where the average age of ACS is 72 years [28, 29], the majority of ACS patients were younger. One possible explanation is that Ethiopia's population is largely made of young people, and CVD is becoming more prevalent among them as a result of lifestyle changes. Unlike previous studies in LMICs that showed males were more likely to be knowledgeable about ACS symptoms [30–34]. Our findings showed that females to be more knowledgeable and is supported by studies conducted in high income countries [24]. The reason for these differences is unclear but may reflect that more highly educated women are living in urban areas such as Addis Ababa and may not reflect women in the general population in Ethiopia. Additional explanations may be that most Ethiopian women are housewives and have a greater opportunity to be exposed to media. Culturally, Ethiopian women are primarily responsible for providing information on health-related issues concerning their family and may have greater interest from this perspective. Other demographic characteristics that predicted ACS knowledge included younger age < 45 years which is supported by several studies conducted in LMICs [35, 36]. From a clinical perspective, diabetic participants were more likely to be knowledgeable about ACS and is supported by several LMIC studies [37]. Conversely, in study elsewhere diabetic patients identified as having lower knowledge of ACS symptoms [38].

Diabetes self-management is complex, and patients are more likely to experience atypical ACS symptoms which can potentially result in poor symptom recognition as other studies have reported [39]. Our findings showed that participants had unfavorable attitudes and beliefs about ACS symptoms. Compared to the report from Jordan by Alfasfos and colleagues (2016), our study participants had slightly lower mean attitude scores (10 vs. 11.5) [40]. Similarly, the finding on the mean belief score was much lower than reported elsewhere [26, 40]. The reasons for these differences are unclear, but may reflect dissimilarity in health care provisions, accessibility and educational level.

According to the European society of cardiology guidelines, patients with ACS symptoms should call the emergency medical system (EMS) or seek treatment as soon as possible, [41] ideally within 10-min of symptom onset to reduce morbidity and mortality. It is estimated that for every 1-min delay to treatment, there is an 2% increase in mortality [42]. Use of EMS is an essential service that has been shown to reduce delay in ACS treatment in LMICs [43]. In the present study, the majority preferred for someone to drive them to the hospital rather than have EMS come to their home. This may be due to a lack of awareness about the existing prehospital system, limited access and fear of delays in transport. In Ethiopia, EMS is underdeveloped and limited to major cities, and individuals are required to pay out of pocket, with no insurance coverage which may further limit accessibility. Currently, there is no coordinated dispatch center in Ethiopia for EMS, the ministry of health however, in collaboration with major urban cities are trying to establish the centers to better support cardiac care in the country [44, 45]. Participants who were exposed to mass media and had adequate social support, had favorable attitudes and beliefs about ACS. This finding clearly identifies exposure to media as a good source for acquiring knowledge about ACS in Ethiopia and needs to be utilized more prominently. This is supported by evidence which identified mass media for enhancing public health education on a variety of topics [46]. An intervention study using mass-media also demonstrated that this format was able to improve knowledge, attitude and beliefs towards ACS in Ireland [9]. Mass media campaigns using radio/television are essential to improve public awareness about ACS, its causes and what actions to take when symptoms occur.

### Strengths

This study was unique by examining knowledge, attitudes and beliefs from currently admitted ED patients diagnosed with ACS. To our knowledge, this is the first study to examine these factors in Ethiopia and provides evidence for the need to implement a national ACS public awareness campaign to reduce future morbidity and mortality. The ACS response Index had adequate psychometric properties instrument and was successfully back translated and used in an Ethiopian population. It will be possible to use this instrument in other segments of the Ethiopian population to compare differences and similarities in knowledge, attitudes and beliefs about ACS. Approximately 35% were female in the study which is much higher than in most LMICs examining ACS. Furthermore, because the study was conducted in a tertiary care hospital, we had easily access to patients who had a confirmed diagnosis of ACS.

### Limitations

Since no study is without limitations, recall bias may have occurred, potentially altering study findings. Participants in the EU were provided a list of ACS symptoms by their treating physician, which may have influenced their responses and resulted in an overestimation of knowledge. Also, because participants were from private and tertiary care hospitals in urban settings, the findings may not be generalizable to community hospitals in rural geographic locations and may not reflect the general Ethiopian community. Many of our study participants were educated and also had a high socioeconomic status, particularly those who went to a private hospital, which may have influenced their responses and may not represent others with lower socioeconomic status.

## Conclusion

Our findings suggest, despite receiving treatment for ACS in an ED, roughly half of participants have inadequate knowledge, attitude and belief towards ACS symptoms. The study finding elucidates the overall all poor health literacy coupled with significant communication gap between the treating health care providers in EU and patients. Contrary to international ACS treatment guidelines, most of our participants did not prefer to use EMS for transport which should be further investigated and addressed. Therefore, our finding showed there is a need to provide health awareness campaigns using different media outlets, focusing on high-risk groups to improve the knowledge attitude and belief of participants towards ACS symptoms. Future large-scale research is also needed to further understand the problem and address the observed gaps.

## Data Availability

The datasets generated and/or analyzed during the current study are not publicly available as public data sharing was not approved by IRB but are available from the corresponding author on reasonable request.

## References

[1] Amsterdam EA (2014). 2014 AHA/ACC guideline for the management of patients with non–ST-elevation acute coronary syndromes: a report of the American College of Cardiology/American Heart Association Task Force on Practice Guidelines. J Am Coll Cardiol.

[2] Roth GA (2020). Global burden of cardiovascular diseases and risk factors, 1990–2019: update from the GBD 2019 study. J Am Coll Cardiol.

[3] Ali S (2021). The burden of cardiovascular diseases in Ethiopia from 1990 to 2017: evidence from the Global Burden of Disease Study. Int Health.

[4] WHO, WHO, W. W. H. F. (2017) WHO | Cardiovascular diseases (CVD) in WHO, W. W. H. F. (2017) WHO | Cardiovascular diseases (CVD) http://www.who.int/mediacentre/factsheets/fs317/en. 2017: http://www.who.int/mediacentre/factsheets/fs317/en.

[5] Abdosh T (2019). Cardiovascular diseases risk factors among adult diabetic patients in eastern Ethiopia. JRSM Cardiovasc Dis.

[6] Tamiru S, Alemseged F (2010). Risk factors for cardiovascular diseases among diabetic patients In Southwest Ethiopia. Ethiop J Health Sci.

[7] Tesfaye, F., Epidemiology of cardiovascular disease risk factors in Ethiopia: the rural-ruban gradient. 2008, Epidemiologi och folkhälsovetenskap.

[8] Nakashima T, Tahara Y (2018). Achieving the earliest possible reperfusion in patients with acute coronary syndrome: a current overview. J Intensive Care.

[9] O'Brien F (2014). Improving knowledge, attitudes and beliefs about acute coronary syndrome through an individualized educational intervention: a randomized controlled trial. Patient Educ Couns.

[10] Darawad MW (2016). Predictors of delay in seeking treatment by Jordanian patients with acute coronary syndrome. Int Emerg Nurs.

[11] Ghazawy ER, Seedhom AE, Mahfouz EM (2015). Predictors of delay in seeking health care among myocardial infarction patients, Minia District, Egypt. Adv Prev Med.

[12] Hadid LAA (2020). Factors associated with prehospital delay among men and women newly experiencing acute coronary syndrome: a qualitative inquiry. Cardiol Res Pract.

[13] McKee G (2013). Multivariate analysis of predictors of pre-hospital delay in acute coronary syndrome. Int J Cardiol.

[14] Gallagher R (2013). A pre-test post-test study of a brief educational intervention demonstrates improved knowledge of potential acute myocardial infarction symptoms and appropriate responses in cardiac rehabilitation patients. Aust Crit Care.

[15] McKinley S (2009). The effect of a short one-on-one nursing intervention on knowledge, attitudes and beliefs related to response to acute coronary syndrome in people with coronary heart disease: a randomized controlled trial. Int J Nurs Stud.

[16] Press, C.U., Cambridge academic dictionary in Cambridge Academic Content Dictionary. 2017, Cambridge University Press.

[17] Jensen LA, Moser DK (2008). Gender differences in knowledge, attitudes, and beliefs about heart disease. Nurs Clin North Am.

[18] Hong MK (2012). Recent advances in the treatment of ST-segment elevation myocardial infarction. Scientifica (Cairo).

[19] Mesas CE (2018). Symptoms awareness, emergency medical service utilization and hospital transfer delay in myocardial infarction. BMC Health Serv Res.

[20] Abdissa SG (2014). Spectrum of cardiovascular diseases among ethiopian patients At tikur anbessa specialized university teaching hospital, Addis ababa. Ethiop Med J.

[21] Cohen L, Manion L, Morrison K (2017). Statistical significance, effect size and statistical power. Research Methods in Education.

[22] Riegel B (2007). Psychometric evaluation of the Acute Coronary Syndrome (ACS) response index. Res Nurs Health.

[23] Albarqouni L (2016). Patients' knowledge about symptoms and adequate behaviour during acute myocardial infarction and its impact on delay time: findings from the multicentre MEDEA Study. Patient Educ Couns.

[24] Dracup K (2008). Acute coronary syndrome: What do patients know?. Arch Intern Med.

[25] Khan MS (2007). High prevalence of lack of knowledge of symptoms of acute myocardial infarction in Pakistan and its contribution to delayed presentation to the hospital. BMC Public Health.

[26] Noureddine S, Dumit NY, Maatouk H (2020). Patients' knowledge and attitudes about myocardial infarction. Nurs Health Sci.

[27] Negesa LB (2020). Patients’ knowledge on cardiovascular risk factors and associated lifestyle behaviour in Ethiopia in 2018: a cross-sectional study. PLoS ONE.

[28] Hasdai D (2002). A prospective survey of the characteristics, treatments and outcomes of patients with acute coronary syndromes in Europe and the Mediterranean basin; the Euro Heart Survey of Acute Coronary Syndromes (Euro Heart Survey ACS). Eur Heart J.

[29] Tang EW, Wong CK, Herbison P (2007). Global Registry of Acute Coronary Events (GRACE) hospital discharge risk score accurately predicts long-term mortality post acute coronary syndrome. Am Heart J.

[30] Allana S (2015). Gender differences in factors associated with prehospital delay among acute coronary syndrome patients in Pakistan. J Transcult Nurs.

[31] Bezdah L (2020). Delays in management of ST-segment elevation myocardial infarction. Arch Cardiovasc Dis Suppl.

[32] Pastorius Benziger C (2011). Sex differences in health care-seeking behavior for acute coronary syndrome in a low income country, Peru. Crit Pathw Cardiol.

[33] Seef S, Jeppsson A, Stafström M (2013). What is killing? People’s knowledge about coronary heart disease, attitude towards prevention and main risk reduction barriers in Ismailia Egypt (Descriptive crosssectional study). Pan Afr Med J.

[34] Xavier D (2008). Treatment and outcomes of acute coronary syndromes in India (CREATE): a prospective analysis of registry data. The Lancet.

[35] Khan A (2017). A study of prehospital delay patterns in acute myocardial infarction in an urban tertiary care institute in Mumbai. J Assoc Phys India.

[36] Lim SC, Rahman A, Yaacob NM (2019). Pre-hospital factors influencing time of arrival at emergency departments for patients with acute ST-elevation myocardial infarction. Malays J Med Sci MJMS.

[37] Mohan B (2018). Factors influencing prehospital delay in patients presenting with ST-elevation myocardial infarction and the impact of prehospital electrocardiogram. Indian Heart J.

[38] Johnson CAH (2021). Knowledge, attitudes, and beliefs about acute coronary syndrome among patients with type 2 diabetes. Rev Lat Am Enfermagem.

[39] Khafaji HAH, Al Suwaidi JM (2014). Atypical presentation of acute and chronic coronary artery disease in diabetics. World J Cardiol.

[40] Alfasfos N (2016). Knowledge, attitudes, beliefs and perceived risk of acute coronary syndrome among Jordanian patients. Health.

[41] Collet J-P (2021). 2020 ESC Guidelines for the management of acute coronary syndromes in patients presenting without persistent ST-segment elevation: the Task Force for the management of acute coronary syndromes in patients presenting without persistent ST-segment elevation of the European Society of Cardiology (ESC). Eur Heart J.

[42] Żurowska-Wolak M (2019). The effects of prehospital system delays on the treatment efficacy of STEMI patients. Scand J Trauma Resusc Emerg Med.

[43] Beza L (2021). Acute coronary syndrome treatment delay in low to middle-income countries: a systematic review. Int J Cardiol Heart Vasc.

[44] Gebru AA, Mosadeghrad AM, Sari AA (2019). Perceptions of leadership, motivation, structure, and assurance for implementation of emergency medical services in Ethiopia: perspectives of emergency medical services case teams based on focus group discussions. Hum Antibodies.

[45] Sultan M (2019). Trends and barriers of emergency medical service use in Addis Ababa; Ethiopia. BMC Emerg Med.

[46] Hanson C (2011). Use and acceptance of social media among health educators. Am J Health Educ.

